# Effect of uphill running on myocardium T_2_ in *mdx* mice

**DOI:** 10.1186/1532-429X-14-S1-P59

**Published:** 2012-02-01

**Authors:** Sean C Forbes, Ravneet S Vohra, Fan Ye, Krista Vandenborne, Glenn A Walter

**Affiliations:** 1 Department of Physical Therapy, University of Florida, Gainesville, FL, USA; 2 Physiology and Functional Genomics, University of Florida, Gainesville, FL, USA

## Background

Cardiac dysfunction is a major cause of death in Duchenne muscular dystrophy. In *mdx* mice, the lack of functional dystrophin localized to the cell membrane leads to increased susceptibility to muscle damage and enhanced muscle degeneration. In this study we examined the effect of an uphill running protocol (Michele et al. Circ Res. 105(10):984-93, 2009) on myocardium transverse relaxation time (T_2_) in young adult *mdx* mice (16 weeks).

## Methods

A 4.7T Oxford Magnet with an Agilent/Varian operating system was used to acquire gated T_2_-weighted single spin-echo images of the left ventricle in the short axis view (TR 750 ms; TE 14-16 ms and TE 30-32 ms; field of view, 25X25 mm2; slice thickness, 1.0 mm; acquisition matrix size, 256 X 128; averages, 8). Images were acquired in C57Bl10 (n=5, male) and *mdx* (n=5, male) mice using a custom built quadrature volume coil (3.3 cm inner diameter). Mice performed uphill treadmill running at a speed of 6-13m/min with a 10 degree incline for up to one hour. MR data were acquired prior to exercise and after 16-24 hrs of exercise. Short axis slices from the mid-papillary region were selected to calculate mean T_2_. Mean T_2_ of the myocardium was calculated using the average signal intensity at each TE by manually tracing the myocardium.

## Results

**Figure 1:**
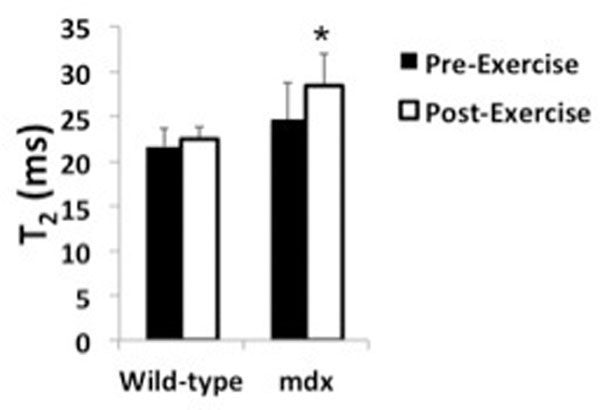
Myocardium T_2_ values before and after uphill running in wild-type and *mdx* mice. *Denotes significantly different than pre-exercise.

Each control mouse completed one hour of uphill running (800 meters), while there was considerable variability in the amount of time and distance run by the mdx mice (33±21(SD) min; 329±227 meters), with only one *mdx* mouse completing one hour. In wild-type mice, there was no effect (p>0.05) of this uphill running protocol on myocardium T_2_ after exercise (Fig. 1). In *mdx* mice, an increase in myocardium T_2_ was observed in each mouse (range: 5 to 32%; Fig. 1). There did not appear to be a direct relationship between running time and T_2_ increase (r=.14, p>0.05).

## Conclusions

The increase in myocardium T_2_ following exercise in *mdx* mice is consistent with dystrophic muscle having an increased susceptibility to muscle damage. Therefore, this *in vivo* exercise protocol monitored using cardiac MRI may be valuable in future studies to test the efficacy of potential therapeutic treatments in dystrophic murine models. Furthermore, this study supports the notion that T_2_ may be valuable for evaluating myocardium involvement in muscular dystrophy.

## Funding

Supported by American Heart Association Post-doctoral Fellowship and Wellstone Muscular Dystrophy Center (1U54RO52646-01A1).

